# Fine-tuned SRF activity controls asymmetrical neuronal outgrowth: implications for cortical migration, neural tissue lamination and circuit assembly

**DOI:** 10.1038/srep17470

**Published:** 2015-12-07

**Authors:** Marilyn Scandaglia, Eva Benito, Cruz Morenilla-Palao, Anna Fiorenza, Beatriz del Blanco, Yaiza Coca, Eloísa Herrera, Angel Barco

**Affiliations:** 1 Instituto de Neurociencias (Universidad Miguel Hernández-Consejo Superior de Investigaciones Científicas). Av. Santiago Ramón y Cajal s/n. Sant Joan d’Alacant. 03550. Alicante, Spain

## Abstract

The stimulus-regulated transcription factor Serum Response Factor (SRF) plays an important role in diverse neurodevelopmental processes related to structural plasticity and motile functions, although its precise mechanism of action has not yet been established. To further define the role of SRF in neural development and distinguish between cell-autonomous and non cell-autonomous effects, we bidirectionally manipulated SRF activity through gene transduction assays that allow the visualization of individual neurons and their comparison with neighboring control cells. *In vitro* assays showed that SRF promotes survival and filopodia formation and is required for normal asymmetric neurite outgrowth, indicating that its activation favors dendrite enlargement versus branching. In turn, *in vivo* experiments demonstrated that SRF-dependent regulation of neuronal morphology has important consequences in the developing cortex and retina, affecting neuronal migration, dendritic and axonal arborization and cell positioning in these laminated tissues. Overall, our results show that the controlled and timely activation of SRF is essential for the coordinated growth of neuronal processes, suggesting that this event regulates the switch between neuronal growth and branching during developmental processes.

The assembly of neuronal networks is accomplished through a precise sequence of developmental processes that are highly dependent on cellular motile functions, including cell migration, axon versus dendrite specification, neurite outgrowth, axon guidance and synaptic targeting. It is thought that the stimulus-regulated transcription factor (TF) Serum Response Factor (SRF) plays a role in these processes by regulating actin dynamics^1^,^2^. SRF binds to a specific DNA sequence referred to as “SRF Response Element” or SRE (also termed “CArG box”), but its transactivation activity depends on the interaction with two families of coactivators, the MRTFs (myocardin-related transcription factors) and the TCFs (ternary complex factors), which are engaged in the Rho/actin and Ras/MAPKs pathways, respectively^3^,^4^,^5^. This position, at the convergence of different activity-regulated signal transduction cascades and upstream of gene programs controlling cytoskeletal changes, pinpoints SRF as an important regulator of complex adaptive responses involving changes of neuronal morphology^6^.

From the early gastrulation defects observed in conventional knockout (KO) mice to the most specific phenotypes found in tissue-restricted conditional mutants, the deficits associated with SRF’s loss-of-function (LOF) have been consistently related to cytoskeletal perturbations^1^,^2^,^7^,^8^. In the nervous system, conditional, late-prenatal deletion of *Srf* impairs tangential cell migration along the rostral migratory stream and causes the ectopic accumulation of progenitor cells in the subventricular zone (SVZ)^9^. Furthermore, *Srf* loss in forebrain neurons abolishes mossy fiber segregation and causes ectopic fiber growth in the hippocampus^10^, as well as hippocampal and cortical lamination defects that affect both the positioning of cell bodies and layer-restricted termination of commissural and mossy fiber axons^11^. More recently, experiments in conditional KO (cKO) mice lacking SRF in neural progenitor cells also revealed deficits in cortical axonal projections, including corticostriatal, corticospinal, and corticothalamic tracts, that were often associated with the loss of the corpus callosum^12^. Of note, the synaptic plasticity defects observed after SRF ablation in the adult brain^13^,^14^,^15^,^16^ might also originate from defective regulation of cytoskeleton dynamics^6^. Although the aforementioned LOF studies have demonstrated that SRF activity is essential for normal brain development, the precise mechanism by which this TF regulates neuronal growth and circuit formation, as well as the consequences of increasing SRF activity during development *in vivo*, are still unknown.

Here, we investigated the cell-autonomous role of SRF in neuronal growth and neurodevelopment by combining *in vitro* and *in vivo* experiments in which SRF activity was bidirectionally manipulated. Our experiments in neuronal cultures reveal that SRF controls the asymmetric outgrowth of neurites favoring primary dendrite enlargement versus branching. In turn, this cellular function influences different neurodevelopmental processes, including neuronal migration, tissue lamination and circuit assembly, which highlights the critical importance of fine-tuned SRF activity in the development of the nervous system.

## Results

### SRF regulates dendritic outgrowth, synaptogenesis and neuroprotection in neuronal cultures

**Figure 1:**
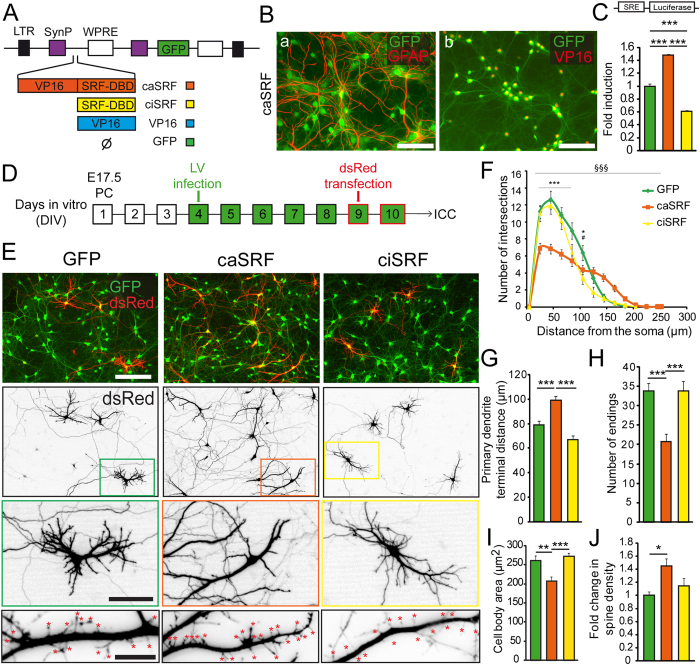
SRF regulates dendritic outgrowth and polarity in neuronal cultures. (**A**) Scheme of lentiviral (LV) constructs and color code used in this study. LTR, Long-termination repeats; SynP, synapsin promoter; WPRE, woodchunk hepatitis virus post-transcriptional regulatory element. (**B)** Hippocampal neurons infected with caSRF were stained for GFP, GFAP and VP16. (a) GFP does not co-localize with the astrocytic specific marker GFAP, (b) Co-expression of VP16 and GFP and presence of VP16-SRF in the nucleus. Scale bar: 100 μm. (**C)** Luciferase reporter assay in HEK293 cells using an SRE-driven luciferase reporter. ***p < 0.0005, 1-way ANOVA followed by Bonferroni’s multiple comparison test (n = 4). (**D)** Scheme of the *in vitro* experiment. PC, primary culture; ICC, immunocytochemistry. (**E)** Hippocampal cultures infected with the different viruses and transfected with dsRed show GFP expression in all neurons (green) and dsRed expression (red) in a small percentage of cells. Scale bar: 100 μm. The two middle rows present representative images of neurons that are both infected and transfected (scale bars: 20 μm). The bottom row shows representative images of dendritic branches in neurons that are both infected and transfected. Scale bar: 5 μm. Neurons expressing caSRF show more filopodia-like processes (red asterisks). (**F**) Sholl analysis presenting number of intersections versus distance to soma. Data are expressed as means ± SEM and compared using 2-way ANOVA followed by Bonferroni’s multiple comparison test. Error bars are shown every 20 μm. The (*) and (#) symbols indicate statistical difference between caSRF and GFP or ciSRF and GFP, respectively; (§) symbols indicate significant interaction between the transduced protein and distance for caSRF or ciSRF and GFP. ^*,#^p < 0.05; ***p < 0.0005; ^§§§^p < 0.0005. (**G**) Quantification of primary dendrite terminal distance. (**H**) Quantification of number of endings. (**I**) Quantification of the cell body area. For **G–I**, **p < 0.005, ***p < 0.0005, 1-way ANOVA followed by Bonferroni’s multiple comparison test. For **F–I**, n = 19–26 in all groups. (**J**) Quantification of synaptic density. *p < 0.05, 1-way ANOVA followed by Bonferroni’s multiple comparison test (n = 9).

To precisely investigate the impact of chronic enhancement or interference of SRF function in neuronal growth, we produced lentivirus-based bicistronic constructs that co-express GFP with different SRF variants under the control of the neuronal-specific synapsin promoter (Fig. 1A). For gain-of-function (GOF) studies, we expressed a constitutively active SRF variant (caSRF) that results from fusing the DNA binding domain (DBD) of SRF with the potent acidic transactivation domain of the viral protein VP16 (Fig. 1B)^17^. For LOF experiments, we expressed a truncated SRF variant that encompasses the DBD of SRF, but lacks any transactivation domain and thereby is expected to act as a competitive inhibitor for SRF binding to its targets (from now referred to as ciSRF)^18^. As control, we used a construct encoding for GFP alone or co-expressing GFP and the VP16 domain (without DBD). Luciferase reporter assays demonstrated the functionality of the SRF-based constructs (Fig. 1C).

We next combined lentiviral infection of primary hippocampal cultures from E17.5 mouse embryos with the transfection of a plasmid encoding dsRed (Fig. 1D). The lower efficiency of transfection compared to infection, together with the ability of dsRed to fill the whole neuron with red fluorescence, allowed a clear visualization of the impact of the chronic manipulation (7 days) of SRF activity on the morphology of individual neurons (Fig. 1E). These experiments led to several conclusions. First, most of the caSRF-infected neurons exhibited bipolar morphology with two or more main/apical dendrites that contrasted with the typical pyramidal shape observed in neurons infected with the control construct (one apical dendrite and several basal dendrites) (Fig. 1E). Furthermore, Sholl analysis evidenced that caSRF neurons present longer but less branched dendrites (Fig. 1F). These observations were confirmed by the quantification of the primary dendrites terminal distance (higher in caSRF neurons, Fig. 1G) and the number of endings (lower in caSRF neurons, Fig. 1H). CaSRF-expressing neurons also had smaller somas (Fig. 1I), which is consistent with the recent observation of enlarged somas in neurons from SRF-deficient mice^19^. Second, the Sholl analysis of ciSRF-transfected cells revealed an overall shape similar to control neurons (GFP) but with slightly reduced dendrite growth and consequently less developed dendritic trees (Fig. 1E–G). This result suggests only a partial interference with endogenous SRF function indicating that SRF/ciSRF heterodimers are likely to retain transactivation activity. Third, caSRF-expressing neurons exhibited a significant increase in spine density (Fig. 1E,J). These spines were thin and filopodia-like indicating that they are likely immature. We did not observe significant changes in spine density in the case of ciSRF-infected neurons. Fourth, despite of the abnormal morphology, neither caSRF nor ciSRF expression were *per se* toxic. Moreover, as previously reported^20^,^21^,^22^, we found that caSRF expression was neuroprotective against excitotoxic insult and serum deprivation (Supplementary Fig. S1).

### SRF regulates dendritic and axonal arborization, as well as neuronal positioning in the developing cortex

**Figure 2:**
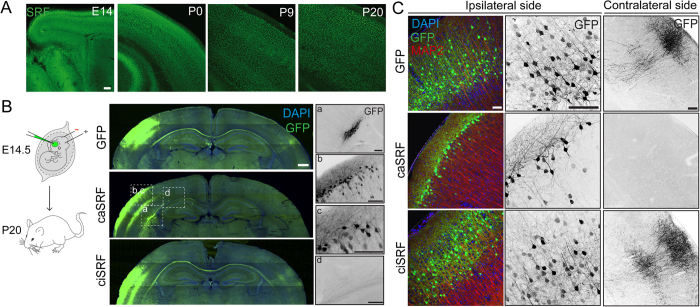
Enhanced SRF activity causes aberrant cell positioning and arborization in the developing cortex. (**A**) Immunostaining against SRF indicates that SRF is expressed during cortex development. Scale bar: 100 μm. (**B**) Left panel: Scheme of the *in utero* electroporation experiment. Center panels: composite images of coronal brain sections of P20 mice electroporated at E14.5 with GFP alone (GFP) or in combination with caSRF or ciSRF. Brain slices were stained with anti-GFP antibody and counterstained with DAPI. Scale bar: 500 μm. Right panels: Four phenotypes associated with caSRF expression: (a) impaired migration of some neurons to the cortical layers. (b) aberrant layer positioning, (c) misoriented apical dendrite, (d) axon mistargeting along corpus callosum. Scale bar: 100 μm. (**C**) Left panels: Higher magnification images of electroporated neurons at the ipsilateral side after triple staining with DAPI (DNA counterstaining), anti-GFP and anti-MAP2 (neuronal dendritic marker). Central and right panels: Detail of GFP staining at the ipsi- and contralateral sides. Scale bar: 100 μm.

SRF is expressed in cortical neurons both during embryonic development and postnatally (Fig. 2A). To investigate its function in neuronal growth and cortical development *in vivo*, we transduced the lentiviral constructs described above in the somatosensory cortex of E14.5 embryos by *in utero* electroporation and examined the brain of electroporated mice at P20 (Fig. 2B). These constructs drove transgene expression *in vivo* as efficiently as in culture. As a result, GFP fluorescence completely filled the cytoplasm of transduced postmitotic neurons, allowing us to analyze dendritic trees in the electroporation site, the crossing fibers at the corpus callosum and axonal arborization at the contralateral side.

Enhanced SRF activity caused four prominent phenotypes (Fig. 2B,C). First, some neurons failed to migrate to the cortical layers and were retained at the SVZ (Fig. 2B-a); second, those cells that migrated were mispositioned along the laminar structure of the cortex and, compared to control GFP-expressing neurons, were much more superficially located (Fig. 2B-b); third, the dendrites of caSRF-expressing cortical neurons were poorly arborized and frequently grew horizontally to the cortical plate instead of projecting an apical dendrite towards the surface as observed in the control condition (Fig. 2B-c); and fourth, whereas the cortico-cortical projections of GFP neurons developed an exuberant arborization in the contralateral side, the axons of caSRF-expressing neurons did not. In fact, some axons were not able to reach their target area in the other hemisphere defasciculating after crossing the midline of the corpus callosum (Fig. 2B-d), which indicates that callosal axon extension and pathfinding are affected in a cell-autonomous manner. In contrast, the expression of ciSRF had a subtler effect in dendrite and axonal arborization than caSRF, although both dendritic ramifications in the ipsilateral side and callosal projections in the contralateral side were thinner and less exuberant than in controls (Fig. 2B,C).

**Figure 3:**
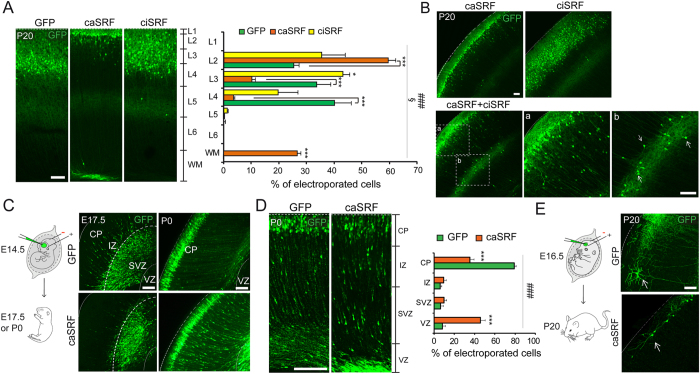
Enhanced SRF activity slows down migration and impairs cortical lamination. (**A**)Quantification of the relative position of the somas of electroporated neurons at P20 in GFP, caSRF and ciSRF conditions revealed the impaired positioning in the cortical layers. The left panels show representative images of electroporated cortices. L1-L6: layer 1- layer 6, WM: white matter (**B**) Partial rescue of caSRF-induced changes in cortical development by co-electroporation with ciSRF. (a) Detail showing the modest impact of ciSRF co-expression in the lamination defect caused by caSRF. (b) The white arrows label multipolar cells-like neurons that were probably halt in the migration process. (**C**) Scheme of the experiment and representative images of coronal brain sections of E17.5 and P0 cortices electroporated at E14.5 with caSRF or GFP. CP: cortical plate, VZ: ventricular zone, IZ: intermediate zone, SVZ: subventricular zone. (**D**) Higher magnification images of P0 cortices. The right bar graph shows the quantification of the relative position of electroporated neurons at P0, showing the delayed migration and accumulation in the VZ of caSRF-expressing neurons. (**E**) Scheme of the experiment and representative images of coronal brain sections (P20) showing the morphology of electroporated neurons located in layer 2 (white arrows). Scale bars: 100 μm. Note that data in bar graphs are expressed as means of the percentage of GFP^+^ cells ± SEM and were compared using 2-way ANOVA for repeated measure, followed by the Bonferroni’s multiple comparison test. The (*) symbols indicate statistical difference between caSRF or ciSRF and GFP control conditions; (#) and (§) symbols indicate statistical interaction between the electroporated construct and layer distribution for caSRF and ciSRF, respectively. *p < 0.05; **p < 0.005; ***p < 0.0005; ^§^p < 0.05; ^###^p < 0.0005 (n = 3).

The first two phenotypes are consistent with impaired radial migration. During cortical development, subsequent waves of newborn neurons migrate along radial glial fibers to form the cortical plate. Each wave of migrating cells travels past its predecessor forming layers in an inside-out manner so that the youngest neurons are the closest to the surface^23^. Therefore, the presence of transduced cells in the SVZ and in the proximity of the cortical surface both indicate that caSRF-expressing neurons have a radial migration defect; either they do not migrate and accumulate at the SVZ or they migrate slower and consequently locate at the most superficial layer. The quantification of the relative position of electroporated neurons along the cortical layers is consistent with this interpretation. Whereas GFP-expressing neurons in control cortices collected at P20 were located across layers 2/3 and 4 (Fig. 3A), migrating caSRF-expressing neurons were found almost exclusively in the upper part of layer 2 (Fig 3A). Interestingly, ciSRF-expressing neurons also located at more superficial positions than control neurons suggesting a slighter impairment in radial migration (Fig. 3A). Further demonstrating the fine sensitivity of radial migration to changes in SRF activity, the co-expression of both SRF variants ameliorated the developmental effects induced by caSRF (Fig. 3B).

To gain additional insight into the effect of chronic cell-autonomous SRF activation during radial migration, we conducted a new series of *in utero* electroporation experiments at E14.5 in which we sacrificed the mice at earlier time points (Fig. 3C). At E17.5, numerous control GFP-expressing cells have already moved to the intermediate zone (IZ), whereas most caSRF-expressing neurons remained in the proximity of the SVZ. At P0, most GFP-expressing neurons in control cortices have reached the cortical plate (CP), while in caSRF-electroporated cortices a lower percentage of labeled neurons has reached this position, with more than 40% of electroporated cells still located at the VZ. These results conclusively demonstrate that radial migration in caSRF-expressing neurons is slower than in control neurons. This migration delay could be explained by the alteration of cell morphology associated with caSRF expression. During radial migration, migrating neurons acquire a characteristic asymmetrical bipolar morphology in which the longer leading process is directed towards the pia and the shorter trailing process is extended below, and failures in acquiring this morphology have been consistently associated with migration defects^24^,^25^.

Intriguingly, these electroporation experiments could also suggest that caSRF expression causes the mispositioning of the soma by mechanisms that are independent of the radial migration defect. A significant percentage of caSRF-expressing neurons have reached the cortical plate at P0 (Fig. 3C–D, ~40%) occupying positions equivalent to those found for electroporated neurons in control cortices. However, caSRF-expressing neurons were still located at the cortical surface 20 days later while, at that time, most control neurons have relocated to inner layers (Fig. 3A), indicating that, after migration, another developmental process might be affected by chronic SRF activation. Numerous studies indicate that the characteristic shape and extent of the dendritic arbor in the neurons of each layer result from the interaction between intrinsic developmental programs and local environmental cues, but relatively little is still known about how dendritic outgrowth influences the final position of the neuronal soma in the cortical plate^24^,^26^,^27^. Like for radial migration, the cortical lamination defect can be a consequence of the abnormal dendritic growth because the underdevelopment of the apical dendrite might prevent the correct positioning of the soma after migration. Consistent with this view, when the electroporation was conducted at E16.5, a time in which control neurons find its final location in layer 2, the soma of many caSRF-expressing neurons located even more superficially than when electroporation occurred at E14.5. Furthermore, in these animals we often observed dendrites that projected parallel to the pial surface (Fig. 3E). Together, these results suggest that defects in dendritic growth/arborization can contribute to the strong lamination defect observed in caSRF-electroporated cortices, although alternative interpretations are also possible. For example, caSRF-expressing neurons might not respond to migration stop signals.

**Figure 4:**
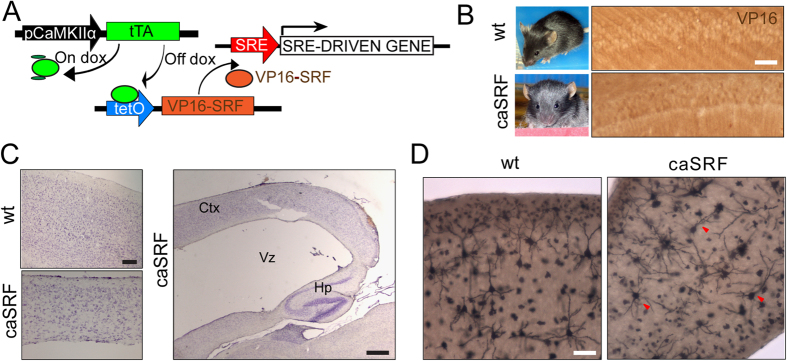
caSRF transgenic mice show severe neurological defects. (**A**) Scheme of the double transgenic approach. (**B**) Images of control and bitransgenic mice (left) and immunostaining of the CA1 subfield using α-VP16 antibody (right). Scale bar: 20 μm (**C**) Nissl staining of wt and caSRF animals. Left panels: Gross abnormalities of cortical somas in caSRF mice. Scale bar: 100 μm. Right panels: Hydrocephalia in caSRF animals. Ctx: cortex, Vz: ventricle, Hp: hippocampus. Scale bar: 500 μm. (**D**) Golgi staining showing the abnormal orientation of apical dendrites in principal cortical neurons (red arrowheads) of caSRF bitransgenics. Scale bar: 100 μm.

Notably, the abnormal orientation of the apical dendrite in neurons expressing caSRF was also observed in a strain of bitransgenic mice generated using the inducible CamKIIα-tTA system (Fig. 4A). In these mice, the expression of caSRF was observed at early-postnatal stages in specific layers of the cerebral cortex and the hippocampus (Fig. 4B) and was associated with severe neurological phenotypes including hydrocephalia (Fig. 4C), reduced body weight and premature death when the animals were 3–4 weeks old (100% of the bitransgenic mice died before reaching 1 month). Golgi staining in brain slices revealed a normal pyramidal morphology in neurons from control mice while those in transgenic mice often had a less polarized morphology (Fig. 4D).

### SRF also regulates lamination, and dendritic and axonal arborization in the visual system

**Figure 5:**
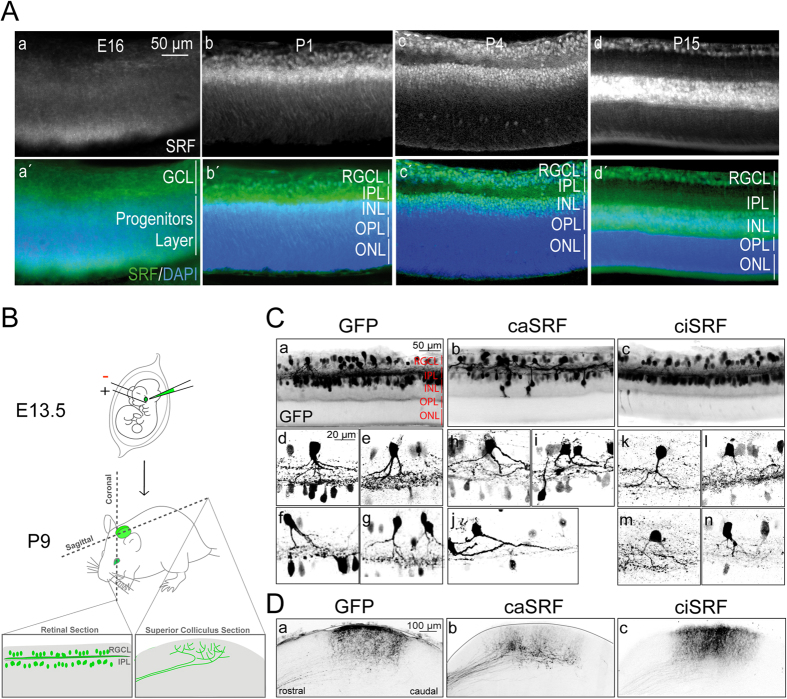
Altered SRF activity also causes growth defects in the developing visual system. (**A**) Expression pattern of SRF in the developing retina at different ages: E16, P1, P4 and P15. (a–d) Immunostaining of SRF in retinal sections from developing or postnatal mice. SRF expression is upregulated after birth. At P4, SRF expression is visible in the retinal ganglion cells and the inner nuclear layers. Two weeks after birth, SRF is still expressed in the RGC layer and strongly expressed in the INL. (a′–d′) Same sections counterstained with DAPI to better visualize the different retinal layers. RGCL, retinal ganglion cells layer; IPL, inner plexiform layer; INL, inner nuclear layer; OPL, outer plexiform layer; ONL, outer nuclear layer. (**B**) Scheme of the experiment. (**C**) SRF modulates retinal dendrites arborization. (a–c) Retinal sections from P9 mice electroporated at E13.5 with plasmids encoding for GFP, caSRF and ciSRF. (d–n) Higher magnification images of retinal neurons from GFP (d–g), caSRF (h–j), or ciSRF (k–n) conditions. (**D**) SRF controls axon branching at the superior colliculus (SC). Sagittal sections through the SC of P9 in the three conditions: GFP (a), caSRF (b) and ciSRF (c).

To determine whether SRF has a similar role in other neural circuits, we turned to the visual system because this is one of the best-established models to investigate dendritic arborization, and axon growth, guidance and targeting^28^. We first analyzed the endogenous expression of SRF in the retina by immunohistochemistry. SRF is expressed in retinal ganglion cells (RGCs) around perinatal stages and its level progressively increases after birth at the time when RGC dendrites grow and axons reach their main target tissues to arborize (Fig. 5A). To specifically transduce RGCs^29^, we next electroporated the retina of E13.5 embryos with the different constructs and sacrificed the pups at P9 (Fig. 5B) when the visual system is nearly mature^28^. GFP fluorescence completely filled the electroporated neurons revealing the dendritic trees in the retina and axonal arborizations in the superior colliculus (SC), which is one of the main targets of retinal projections.

In the control GFP-electroporated retinas, neurons located in the RGC layer had a prominent basal dendrite that separates the soma from the inner plexiform layer (IPL) (Fig 5C, panels a, d–g). However, caSRF-expressing neurons frequently showed more than a principal dendrite emerging parallel to the IPL and concomitant mispositioning of the soma. Furthermore, the dendrites of caSRF-expressing neurons occupied larger areas but exhibited fewer ramifications than the controls (Fig. 5C, panels b, h–j). Although the axons of both GFP- and caSRF-expressing RGCs reached the corresponding topographical area in the SC, the first ones developed exuberant arborization in the target area, whereas the second ones exhibited a grainier appearance, did not arborize properly and never reached the surface of the SC (Fig. 5D, panels a, b). In conclusion, like in the developing cortex, caSRF expression caused lamination defects and the mislocation of somas in the retina. Also consistent with our observations in the cortex, the dendrites in ciSRF-electroporated RGCs seemed thinner than in GFP-expressing control neurons (Fig. 5C, panels c, k–n), and their axons developed smoother arborization at the SC than those of control neurons, although axonal pathfinding was apparently not affected (Fig. 5D, panel c).

### SRF drives the expression of genes related to neuronal growth polarity

**Figure 6:**
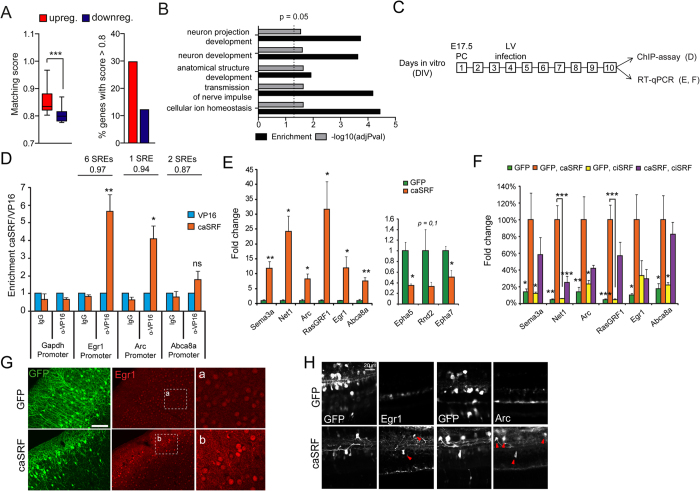
SRF regulates a gene program involved in neurite growth and polarity. (**A**) Left panel: distribution of SRE site prediction scores (>0.8) for SRF-upregulated genes and an equal number of SRF downregulated genes. Right panel: percentage of genes with an SRE matching score >0.8 within up- and downregulated genes. (**B**) Salient GO categories enriched in SRF-upregulated genes. Depicted are the enrichment over baseline and the transformed adjusted p-value. The vertical line indicates the 0.05 significance threshold. (**C**) Scheme of the experiments. Cultures infected at DIV4 and samples collected at DIV10. (**D**) ChIP assays confirm the direct binding of caSRF to the promoter of Arc and Egr1. ChIP-qPCR assays for specific promoters using primers close to the SRE-containing region. The number of SRE sites and the highest Transfac matrix similarity score for SRF binding (obtained with PSCAN) are indicated. α-VP16: specific antibody, IgG: preimmune. *p < 0.05; **p < 0.005: ns: not significant; Student’s t test (n = 4–6). (**E**) RT-qPCR assays confirmed the upregulation (left) and downregulation (right) of candidate genes retrieved in the transcriptomics analysis. *p < 0.05, **p < 0.005 referred to GFP. Student’s t test (n = 3–4). (**F**) The co-infection of neuronal cultures with ciSRF and caSRF shows that ciSRF interferes with caSRF-dependent transcription. Data in bar graphs are expressed as means of the fold changes ± SEM and were compared using 1-way ANOVA, followed by Bonferroni’s multiple comparison test. The (*) symbols indicate statistical difference between all the conditions and caSRF; *p < 0.05; **p < 0.005; ***p < 0.0005 (n = 3–4). (**G**) Immunohistochemistry against Egr1 in cortices of P20 mice electroporated with GFP alone (GFP) or in combination with caSRF (caSRF). Scale bar: 100 μm. (**H**) GFP- and caSRF-electroporated retina showing cells with upregulated Egr1 and Arc upon caSRF expression (red arrowheads).

In order to identify candidate effector genes downstream of SRF responsible for these phenotypes, we turned to the transcriptomics screen presented in^17^. This screen showed that caSRF induces a gene program in hippocampal cultures that is remarkably enriched for SRE motifs (Fig. 6A) at promoter sequences^17^ and for genes related to synaptic transmission, axon guidance and neuronal growth according to Gene Ontology (GO) classification of biological processes (Fig. 6B). This set included genes involved in neuronal growth, such as those encoding the RAS protein-specific guanine nucleotide-releasing factor (Rasgrf1), the neuroepithelial cell transforming protein 1 (Net1), and the semaphorins Sema3a, Sema3d and Sema7a. Intriguingly, although the analysis of TF binding sites enrichment did not reveal enrichment for SRE sites among downregulated genes, this small gene set was also enriched for axon guidance and cytoskeleton remodeling genes, including *Slit2*, *Dcc*, *Nrp1*, *Rnd2, Alcam*, *Epha7*, *Epha3* and *Epha5*.

To examine whether some of these upregulations were the result of the direct binding of caSRF to the gene promoter, we conducted chromatin immunoprecipitation (ChIP) assays in infected hippocampal cultures that confirmed the direct binding to the *Arc* and *Egr1* promoters (Fig. 6C,D). For other candidate genes, we did not detect binding to the predicted regulatory sites, suggesting that either caSRF binds to more distant sites or the regulation is indirect. In parallel, RT-qPCR experiments confirmed the upregulation and downregulation of relevant candidate genes in caSRF-transduced cultures (Fig. 6E). Consistent with our functional assays (Fig. 3B), these experiments also showed that caSRF had a stronger effect in gene expression than ciSRF, although ciSRF counteracted the transcriptional changes promoted by caSRF when both variants were co-expressed (Fig. 6F). Finally, also supporting the consistency between in culture and *in vivo* results, immunohistochemistry analyses in electroporated mice confirmed the upregulation of relevant candidate genes, such as *Egr1*, after ectopic expression of caSRF in cortical neurons (Fig. 6G) and RGCs (Fig. 6H).

## Discussion

We demonstrate here that the manipulation of SRF activity in neurons has important consequences in cell morphology, survival and growth *in vitro* and in the formation of neuronal circuits *in vivo*. Our results are congruent with previous LOF studies in SRF deficient mice indicating that interfering with SRF function alters neuronal growth and neurodevelopment^10^,^11^,^12^ and with *in vitro* GOF experiments indicating that enhancing SRF function promotes neuronal growth^10^,^11^,^21^,^30^ and protects against insults^20^,^21^,^22^. In addition, our GOF experiments using different approaches (viral transduction, in utero electroporation and transgenesis) revealed for the first time a role for SRF regulating asymmetrical outgrowth: caSRF expression favored primary dendrites enlargement versus branching both *in vitro* and *in vivo*. Axonal arborization was also severely reduced both in cortex and retina. This altered neuronal polarity, in turn, affected neuronal migration and the correct positioning of the soma in laminated structures like the cortical plate and the retina. Consistent with the cell-autonomous effect of caSRF expression reported here, recent studies have shown that ectopic expression of a different constitutive active form of SRF caused the mislamination of the photoreceptor layer in the retina of young transgenic animals^31^, whereas SRF deficient mice exhibited defects in hippocampal and cortical lamination^11^ and craniofacial development defects associated with cell migration deficits^32^. How can LOF and GOF manipulations produce similar phenotypes? We hypothesize that complex processes, such as circuit formation and tissue lamination, require fine-tuned SRF activity and are similarly affected by both positive and negative manipulations. Unbalanced or extemporaneous SRF activity both cause growth and arborization defects in a cell-autonomous manner, indicating that caution is needed in evaluating restorative strategies in the nervous system targeted to this TF^30^.

**Figure 7:**
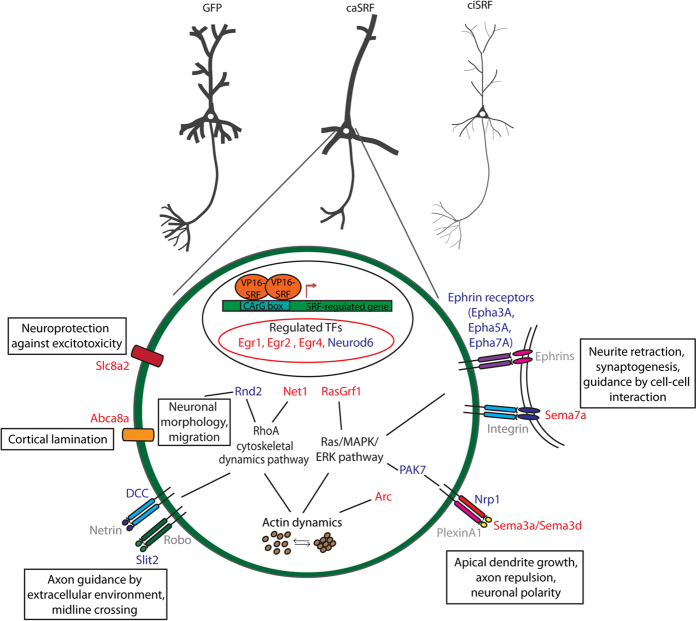
SRF target genes and biological processes affected by caSRF. Scheme highlighting SRF target genes and biological processes. Putative effector proteins upregulated or downregulated upon caSRF expression are shown in red or blue, respectively. Non affected proteins in our transcriptomics analysis are shown in grey.

Our recent transcriptomics analysis^17^ retrieved important candidate genes that were validated here (Fig. 6), and contribute to explain how SRF can regulate the affected processes. In addition to previously reported SRF targets, such as the immediate early gene (IEG) *Arc*, transgelin (*Tagln*) and several members of the *Egr* family (*Egr1*, *Egr2* and *Egr4*), we identified a number of novel downstream genes related to the various phenotypes associated with enhanced SRF-driven transcription (Fig. 7). Thus, among the gene expression changes identified in our transcriptomics screen, we find the downregulation of the ephrins receptors *Epha7*, *Epha3* and *Epha5* and the upregulation of the semaphorin genes *Sema3a*, *Sema3d* and *Sema7a*, all of which are known to play a critical role regulating neuronal migration and/or growth. Of note, experiments in forebrain-restricted *Srf* cKO mice had already suggested that the dysregulation of Ephs and Semaphorin guidance cues might contribute to the reduction of neurite outgrowth and to mossy fiber segregation defects^10^,^33^. In particular, the downregulation of ephrin receptors observed in caSRF-expressing cells might reduce the sensitivity to ephrins and cause the mistargeting of axons observed in our *in utero* experiments, while changes in the expression of semaphorins can provide an explanation for the axon mistargeting and lamination defects^34^. For instance, Sema3a can function as either a chemorepulsive agent, inhibiting axonal outgrowth, or as a chemoattractive agent, stimulating the growth of apical dendrites^35^,^36^. Less is known about Sema7a, but this protein has been shown to promote axonal growth in the embryonic olfactory bulb^37^, and spreading and dendrite outgrowth in melanocytes^38^. Interestingly, the third altered semaphorin, Sema3d, which is known to induce the collapse and paralysis of neuronal growth cones, binds to neuropilin 1 which was also found altered (downregulated) in caSRF-expressing neurons^39^.

Other genes whose function in neurons is less understood can also contribute to the altered morphology. For instance, the downregulation in caSRF-expressing neurons of the small Rho GTPase encoded by *Rnd2* suggests the activation of a homeostatic response to refrain SRF activation. Interestingly, it has been demonstrated that Rnd2 is essential in controlling the multipolar to bipolar transition during the migratory process of pyramidal neurons in the cortex^40^, and *in utero* electroporation of Rnd2 shRNA in the cortex causes migration and lamination defects similar to those reported here for caSRF^41^. Another interesting candidate is Net1, a RhoGEF protein that regulates cytoskeleton dynamics. Intriguingly, exon-level analysis of transcriptomics data revealed that the upregulation of *Net1* in caSRF-expressing neurons was exclusively caused by the overexpression of a shorter, N-terminally truncated variant known as Net1A (Supplementary Fig. S2), which localizes in the cytosol and is thought to stimulate actin polymerization more efficiently than the full-length protein^42^,^43^. In turn, Na^+^/Ca^2+^ exchanger (NCX) encoded by *Slc8a2*, a low affinity, high capacitance calcium anti-porter membrane protein that can switch to ‘reverse mode’ under excitotoxicity and other forms of cellular stress, is a likely candidate to mediate the neuroprotective effect of caSRF^44^. Of note, *Slc8a2* and *Abca8a* (which encodes a lipid transporter and is also upregulated) are two of the genes specifically expressed in the cortical subplate at E15.5^45^, suggesting that their overexpression may contribute as well to the abnormal cortical lamination.

In conclusion, although the list of putative transcriptional targets and their biological role are highly consistent with the phenotypes associated with SRF manipulations, the direct binding to most of these targets remains to be determined and unspecific caSRF-DNA interactions are not excluded. It is expected that several effector genes downstream of SRF redundantly or synergically contribute to the alterations associated with enhanced SRF activity. Functional assays for each of these candidate genes overexpressed either individually or in combination would be required to determine the precise molecular underpinning of the different phenotypes described here.

Little is still known about how the precise regulation of neurite outgrowth by intrinsic and extrinsic signals leads to the intricate branching pattern unique to each neuronal class observed *in vivo*. During development, dendrites and axons of central nervous system neurons grow and refine by addition and retraction of thin branches. This process, reproduced in neuronal cultures, is highly dynamic and only a small fraction of newly added branches remain as part of the mature dendritic and axonal trees. It is thought that the growth rates of axons and dendrites result from the interplay between pre-programmed TFs levels and intrinsic length-sensing mechanisms^46^. Transcription is now thought to primarily occur in the form of pulsatile bursts that rely on continuous oscillations in the expression level or subcellular localization of TFs^47^. Such oscillatory activity is likely disrupted by the expression of a constitutively active variant like caSRF. Pulsatile bursts of SRF activity during neuronal morphogenesis may regulate the asymmetric outgrowth of neuronal processes by balancing enlargement versus branching, thereby providing a link between genome expression and environment-regulated neuronal growth. This view helps to understand how SRF can influence a variety of neurodevelopmental processes, from neuronal migration and tissue lamination to circuit assembly.

## Materials and Methods

### Lentiviral production

To generate the caSRF variant, the DNA binding domain (DBD) of SRF was fused with the acidic transactivation domain of viral protein 16 (VP16) of herpes simplex virus^48^. To produce the ciSRF variant, we expressed the DBD of SRF alone^18^. The VP16-expressing construct, used as control in ChIP assay experiments, carries the transactivation domain of VP16 and lacks any DBD^17^. Both constructs were cloned into the synapsin promoter-bearing lentiviral vector LenLox 3.7^49^ and used either in electroporation experiments or in the production of lentiviral pseudovirions as described^17^. Viral stocks were tittered by RT-qPCR and concentrated through ultracentrifugation.

### Mouse strains

TetO-caSRF transgenic mice were generated by microinjection of the linear construct as previously described^50^. Analysis of transgenic and founder mice was performed by Southern blotting using a VP16 probe. The founder mice were backcrossed to C57BL6/J mice to generate the transgenic line used in our study. We designated as caSRF mice those bitransgenic animals resulted of the crossing of pCaMKII-tTA mice (line B)^50^, and tetO-VP16-SRF transgenics and as wild-type mice those littermates carrying either pCaMKII-tTA, tetO-VP16-SRF or none transgene. Note that the tetO-caSRF transgenic strain is no longer available. The observations reported here are based on the analysis of the bitransgenic mice born in 2005 (5 out of 5 mice showed hydrocephalus). Albino ICR mice were used for primary neuronal culture and *in utero* electroporation experiments in the cerebral cortex, whereas B6D2F1/J mice were used for *in utero* electroporation experiments in the retina because pigmented eyes are easier targets for DNA injection. All mice were maintained according to animal care standards and experimental protocols were approved by the Institutional Animal Care and Use Committee.

### Culture, infection and transfection of hippocampal neurons

Primary hippocampal neurons were obtained from E17.5–E18.5 embryos. Hippocampi were dissected and processed as described^17^. The day of plating was considered day *in vitro* 1 (DIV1). Neurons were incubated for at least 3 additional days (DIV4) before they were used in any experiment. Primary hippocampal neurons were infected at the times indicated by adding the necessary volume of the concentrated viral preparation to achieve an effective multiplicity of infection of 1–10, which provided a percentage of neuronal infection close to 100%. In co-infection experiments, equal volumes of each virus were mixed. In transfection experiments, the plasmid pDsRed-Express2-C1 (Clontech 632538) driving dsRed expression was transfected using Lipofectamine 2000 (Invitrogen) as described by the manufacturer.

### Cell culture assays and treatments

For luciferase assays, HEK293 cells were grown in 90% DMEM 10% fetal calf serum supplemented with 2 mm glutamine and penicillin/streptomycin (100 U/ml to 100 μg/ml) (Invitrogen). HEK293 cells were transfected using Lipofectamine 2000 (Invitrogen) with efficiency larger than 90%. Plasmids were transfected in a ratio of 500:50:1 (construct of interest: Firefly reporter: Renilla reporter) with a total DNA amount of 1 μg per 100.000 cells. The Firefly and Renilla luciferase reporter plasmids used in this study were pSRE-Luc (Clontech 631911) and pRL-SV40 (Promega E2231), respectively.

Luciferase activity was measured at 24 h after transfection, using the Dual Luciferase Assay kit (Promega) as reported before^17^. For cell death assays, primary hippocampal neurons infected at DIV4 were treated with different concentrations of NMDA at DIV9. After 12 hours, a LDH assay was performed using the Cytotoxicity Detection Kit Plus (LDH) (Roche) following manufacturer instructions. For B27 deprivation, complete maintaining media was substituted at DIV7 by B27-deprived media.

### *In utero* electroporation in retina and cortex

Cortex *in utero* electroporations were performed in timed*-*pregnant ICR mice anesthetized with isofluorane; their abdominal cavity was cut open, and the uterine horns exposed. Approximately 1–2 μl of DNA solution (1 μg/μl) was injected into the lateral ventricle of E14.5 or E16.5 embryos using a pulled glass micropipette. In co-electroporation experiments we used the same amount of total DNA (i.e., the concentration of the individual plasmids was 0.5 μg/μl). Each embryo was placed between tweezers-type electrodes (CUY650-P5, NEPA Gene). The angle of inclination of the electrode paddles with respect to the horizontal plane of the brain was zero for targeting the cortical ventricular zone (VZ). Square electric pulses (45 V; 50 ms) were passed five times at 950 ms intervals using an electroporator (CUY21; NEPA Gene). Retina *in utero* electroporation experiments were performed in E13.5 B6D2F1/J embryos as described in^29^. Each experiment was repeated 7 times for a total of 13–18 retinas and SC analyzed. In both cases, only a few embryos were exposed outside the abdominal cavity at a time to prevent excessive temperature loss. The wall and skin of the abdominal cavity were sutured-closed, and embryos were allowed to develop normally till E17.5, P0, P9 or P20.

### Immunocytochemistry and immunohistochemistry

Immunostainings in primary hippocampal cultures were conducted as described in^17^. For Sholl analysis, the dendrites of transfected neurons were drawn and analyzed with Neurolucida neuron tracing Software (MBF Bioscience) in a blind manner. The number of intersections was determined every 2 μm, starting from a radius of 5 μm to the center of the neuronal soma. For immunohistochemistry, mice were perfused with 4% paraformaldehyde in PBS. Coronal vibratome sections (50 or 100 μm) were obtained from electroporated brains; washed in PBS and PBS−0.1% Triton X-100 (PBT) and incubated for 0,5 h at room temperature with 3% BSA-PBT. The primary antibodies used in this study are α-GFP (Aves Labs, GFP-1020), α-MAP2 (Sigma, M9942), α-GFAP (Sigma-Aldrich, G9269), α-VP16 (mouse monoclonal from Santa Cruz Biotechnology sc-7545; rabbit polyclonal from Sigma-Aldrich V4388), α-Egr1 (Santa Cruz Biotechnology, sc-110), α-SRF (Santa Cruz Biotechnology sc-335), α-dsRed (Clontech, 632496) and α-Arc (Synaptic Systems, 156-003). Nuclei were counterstained with a 1 nM DAPI solution (Invitrogen) before mounting. Images were taken with Olympus Confocal Inverted Microscope or a Leica epifluorescence Microscope.

### RNA extraction, RT-qPCR and microarray analysis

Total RNA from hippocampal cultures was extracted with TRI reagent (Sigma-Aldrich) and reverse transcribed to cDNA using the RevertAid First-Strand cDNA Synthesis kit (Fermentas). qPCR was performed in an Applied Biosystems 7300 real-time PCR unit using Eva Green qPCR reagent mix. The primer sequences used in the RT-qPCR assays are shown in Supplementary Table 1. Each independent sample was assayed in duplicate and normalized using GAPDH levels. New bioinformatical analyses were performed using the datasets presented in^17^ and accessible at ArrayExpress database (accession number E-MEXP-3167). The enrichment for TFBS was calculated with PSCAN using JASPAR (Fig. 6A) or Transfac (Fig. 6D) as the reference databases, and exploring from −950 to +50 bp region of promoters^51^. Webgestalt^52^ was used to perform the GO analysis of caSRF-regulated gene (FDR 0.1, >2 genes per category).

### Chromatin immunoprecipitation assays

Chromatin immunoprecipitation (ChIP) experiments were conducted as previously described^53^ with some modifications. Primary hippocampal neurons infected with caSRF or VP16 at DIV4 were washed with PBS at DIV10, and cross-linked by the addition of 1% formaldehyde (v/w) and incubated for 10 min at room temperature. The reaction was stopped by adding glycine to 0.125 M and incubating for 5 min at RT. After two washes in PBS, neurons (2 × 10^6^) were scraped and resuspended in 400 μl 1% SDS, 10 mM EDTA, 50 mM Tris-HCl (pH 8.1), and protease inhibitors and incubated for 10 min on ice. The cell suspension was sonicated using a Branson sonicator at 10% of capacity, 6 cycles of 15 sec. Chromatin was used for ChIP by incubation for 16 h at 4 °C with 5 μg specific anti-VP16 (from Sigma V4388) or isotype-matched controls Abs, followed by 2.5 h incubation at 4 °C with 30 μl protein G Dynabeads. The DNA was resuspended in 30 μl of water for subsequent PCR analysis as a template to evaluate the presence of regulatory regions of caSRF targets. The primer sequences used in the ChIP-qPCR assays are listed in Supplementary Table S1. Paired sample *t*-tests were used to determine the statistical significance between values.

## Additional Information

**How to cite this article**: Scandaglia, M. *et al.* Fine-tuned SRF activity controls asymmetrical neuronal outgrowth: implications for cortical migration, neural tissue lamination and circuit assembly. *Sci. Rep.* **5**, 17470; doi: 10.1038/srep17470 (2015).

## Supplementary Material

Supplementary Information
