# Supplementary material for: Fine-tuned SRF activity controls asymmetrical neuronal outgrowth: implications for cortical migration, neural tissue lamination and circuit assembly
Source: Sci Rep. 2015 Dec 7;5:17470. doi: 10.1038/srep17470 (PMC4671020; doi:10.1038/srep17470)
Supplement: Supplementary Information [file srep17470-s1.pdf]

**SUPPLEMENTARY INFORMATION**

**Title:** Fine-tuned SRF activity controls asymmetrical neuronal outgrowth: implications for cortical migration, neural tissue lamination and circuit assembly

**Authors:** Marilyn Scandaglia, Eva Benito, Cruz Morenilla-Palao, Anna Fiorenza, Beatriz del Blanco, Yaiza Coca, Eloísa Herrera and Angel Barco

**Inventory of supplementary information:**

• Legend for Supplementary Figures S1 and S2.

• Supplementary Figure S1. caSRF promotes neuronal survival.

• Supplementary Figure S2. Net1A is selectively upregulated in caSRFexpressing cultures.

• Supplementary Table S1. Primers used in this study.

**SUPPLEMENTARY FIGURE LEGEND**

**Supplementary Figure S1. caSRF promotes neuronal survival. A.** Scheme of the experiment and quantification of LDH activity in LV-infected cultures at different NMDA concentrations. caSRF expression ameliorates NMDA-induced neurotoxicity (n = 9). **B.** Scheme of the experiment and quantification of LDH activity after 7 days of trophic deprivation. caSRF expression also reduces neuronal death induced by serum deprivation indicated as -B27 (n = 8). MM: complete maintaining media. #, *: $p < 0.05$; ##: $p < 0.005$, Student's t-test (* symbol used for statistical difference between lentiviral vectors infected cultures, # symbol used for statistical difference between treatments).

**Supplementary Figure S2. Net1A is selectively upregulated in caSRF-expressing cultures.** Left: Exon-level analysis of microarray data for the Net1 gene. Right: RT-qPCR confirmed the specific upregulation of the short variant Net1A.

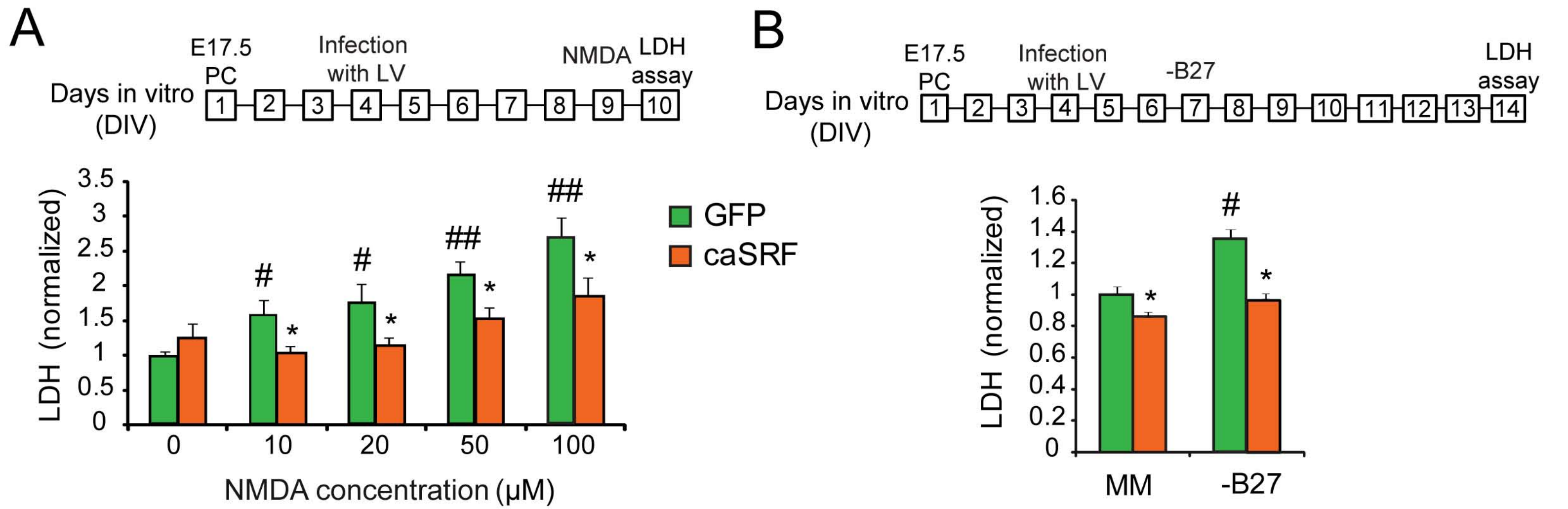
A
E17.5
PC
Infection
with LV
NMDA
LDH
assay
Days in vitro
(DIV)
1
2
3
4
5
6
7
8
9
10
LDH (normalized)
3.5
3
2.5
2
1.5
1
0.5
0
#
#
##
##
*
*
*
*
0
10
20
50
100
NMDA concentration (μM)
GFP
caSRF
B
E17.5
PC
Infection
with LV
-B27
LDH
assay
Days in vitro
(DIV)
1
2
3
4
5
6
7
8
9
10
11
12
13
14
LDH (normalized)
1.6
1.4
1.2
1
0.8
0.6
0.4
0.2
0
#
*
*
MM
-B27

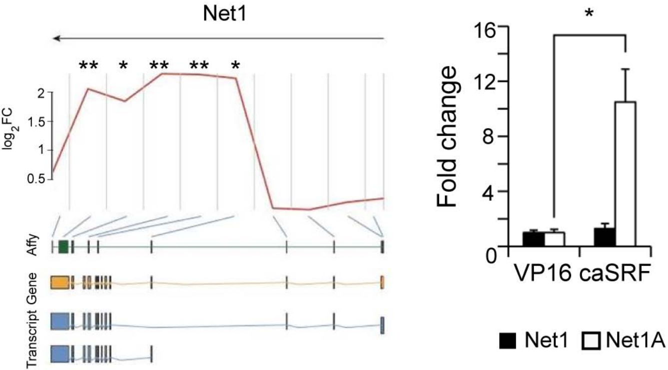
Net1
** * ** ** *
2
1.5
1
0.5
log2FC
Affy
Transcript Gene
*
16
12
8
4
0
Fold change
VP16 caSRF
Net1 Net1A

**Supplementary Table S1: Primers used in this study.**

Primer sequences used in the RT-qPCR

| | Forward (5'-3') | Reverse (5'-3') |
|---|---|---|
| Sema3a | CTTACAGCCAGAGCAAACTATG | GAAGGAAGGTGTGGTAACTGG |
| Net1 | AAGGACGATCCGAGTCTTAGATG | AGCTAGAGGCCGAACTCTCTTATTG |
| Arc | GCAGGAGAACTGCCTGAACAG | AAGACTGATATTGCTGAGCCTCAA |
| RasGRF1 | GACCTTGGAGTTGAGGACCG | CGCCTCATGTTCTGTAGCCA |
| Egr1 | CCGTCCTGTTCCCTTTGACTT | GAACTTGGACATGGCTGTTTCAG |
| Abca8a | GGGGTCATATTCACCAACGC | TGAGCTGTGTGGTCCTGATG |
| Epha5 | ACCTTGGAGGATTTGAGGCG | TCCCGTTTACCATCTGCACC |
| Rnd2 | AAGATCGTAGTGGTGGGGGA | GATCTCGAAGCTGGCAGTGT |
| Epha7 | GGTGTTCATGGTGTTCGGCT | AACTGACAGGTGCTCATTTGTTAC |
| Gapdh | CTTCACCACCATGGAGAAGGC | CATGGACTGTGGTCATGAGCC |

Primer sequences used in the ChIP-qPCR

| | Forward (5'-3') | Reverse (5'-3') |
|---|---|---|
| Arc | TGTTGCCAGGGAATCGGAAG | GCAGAGGAAAGCAAGATGCC |
| Egr1 | TTGGATGGGAGGGCTTCAC | CTCCGCCGTGACGTACAT |
| Abca8a | GAGCTCAGAGCCTTTTAAGGGA | CCTCCCCCAAAAGAAATTGACAG |
| Gapdh | TTCACCTGGCACTGCACAA | CCACCATCCGGGTTCCTATAA |
